# RETRACTED: Kim et al. The Angiogenesis Inhibitor ALS-L1023 from Lemon-Balm Leaves Attenuates High-Fat Diet-Induced Nonalcoholic Fatty Liver Disease Through Regulating the Visceral Adipose-Tissue Function. *Int. J. Mol. Sci.* 2017, *18*, 846

**DOI:** 10.3390/ijms27135852

**Published:** 2026-06-29

**Authors:** Jeongjun Kim, Haerim Lee, Jonghoon Lim, Jaeho Oh, Soon Shik Shin, Michung Yoon

**Affiliations:** 1 Department of Biomedical Engineering, Mokwon University, Daejeon 35349, Republic of Korea; sdsd2586@naver.com (J.K.); lhr6515@naver.com (H.L.); tbvjwhdtlsdl@naver.com (J.L.); kelberoth30@naver.com (J.O.); 2 Department of Formula Sciences, College of Korean Medicine, Dongeui University, Busan 47340, Republic of Korea; ssshin@deu.ac.kr

The journal retracts the article titled “The Angiogenesis Inhibitor ALS-L1023 from Lemon-Balm Leaves Attenuates High-Fat Diet-Induced Nonalcoholic Fatty Liver Disease through Regulating the Visceral Adipose-Tissue Function” [1], cited above.

Following publication, concerns were brought to the attention of the Editorial Office regarding the integrity of a figure presented in this article [1].

In accordance with the journal’s standard procedures, an investigation was conducted by the Editorial Office and Editorial Board which identified image duplication with figures previously published [2] by a similar authorship group without attribution or disclosure. Despite multiple attempts to contact the authors and their affiliated institution, no response was received. In the absence of authors’ clarification and access to the underlying data necessary to verify the reported results, the Editorial Board has lost confidence in the integrity and reliability of the findings. Consequently, this publication has been retracted according to MDPI’s retraction policy.

This retraction was approved by the Editor-in-Chief of the *International Journal of Molecular Sciences*.

The authors have been informed of this retraction but did not provide a comment on this decision.

## References

[1] Kim J., Lee H., Lim J., Oh J., Shin S.S., Yoon M. (2017). RETRACTED: The Angiogenesis Inhibitor ALS-L1023 from Lemon-Balm Leaves Attenuates High-Fat Diet-Induced Nonalcoholic Fatty Liver Disease Through Regulating the Visceral Adipose-Tissue Function. Int. J. Mol. Sci..

[2] Park B.Y., Lee H., Woo S., Yoon M., Kim J., Hong Y., Lee H.S., Park E.K., Hahm J.C., Kim J.W. (2015). Reduction of Adipose Tissue Mass by the Angiogenesis Inhibitor ALS-L1023 from *Melissa officinalis*. PLoS ONE.

